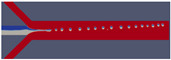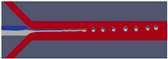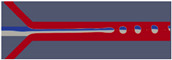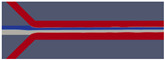# Generation and Dynamics of Janus Droplets in Shear-Thinning Fluid Flow in a Double Y-Type Microchannel

**DOI:** 10.3390/mi12020149

**Published:** 2021-02-03

**Authors:** Fan Bai, Hongna Zhang, Xiaobin Li, Fengchen Li, Sang Woo Joo

**Affiliations:** 1 School of Mechanical Engineering, Tianjin University, Tianjin 300072, China; baifan@ynu.ac.kr (F.B.); lixiaobin@tju.edu.cn (X.L.); lifc@tju.edu.cn (F.L.); 2 School of Mechanical Engineering, Yeungnam University, Gyeongsan 38541, Korea

**Keywords:** microfluidics, Janus droplet, OpenFOAM, volume of fluid method, adaptive dynamic mesh refinement, shear-thinning fluid

## Abstract

Droplets composed of two different materials, or Janus droplets, have diverse applications, including microfluidic digital laboratory systems, DNA chips, and self-assembly systems. A three-dimensional computational study of Janus droplet formation in a double Y-type microfluidic device filled with a shear-thinning fluid is performed by using the multiphaseInterDyMFoam solver of the OpenFOAM, based on a finite-volume method. The bi-phase volume-of-fluid method is adopted to track the interface with an adaptive dynamic mesh refinement for moving interfaces. The formation of Janus droplets in the shear-thinning fluid is characterized in five different states of tubbing, jetting, intermediate, dripping and unstable dripping in a multiphase microsystem under various flow conditions. The formation mechanism of Janus droplets is understood by analyzing the influencing factors, including the flow rates of the continuous phase and of the dispersed phase, surface tension, and non-Newtonian rheological parameters. Studies have found that the formation of the Janus droplets and their sizes are related to the flow rate at the inlet under low capillary numbers. The rheological parameters of shear-thinning fluid have a significant impact on the size of Janus droplets and their formation mechanism. As the apparent viscosity increases, the frequency of Janus droplet formation increases, while the droplet volume decreases. Compared with Newtonian fluid, the Janus droplet is more readily generated in shear-thinning fluid due to the interlay of diminishing viscous force, surface tension, and pressure drop.

## 1. Introduction

Droplet-based microfluidic technology has many advantages in biomedicine, chemical analysis, material science and microreactions due to its capability to produce high surface-volume ratios in large quantities with low reagent use, rapid reaction, and independent control of each droplet [1,2,3,4,5,6,7]. Many studies have been carried out to develop efficient and stable methods for conventional single-phase and multiphase droplet formation and movements [8]. Janus droplet, composed of two adhering immiscible drops of different fluids in a third phase, can offer a wide range of applications that cannot be realized with single-attribute structures, due to their centrally asymmetrical structure. The flow mechanism of Janus droplets in a microfluidic environment is diverse, including wettability gradient, magnetic force, and electrical force, among others [9,10]. The formation of bi-phase structures of droplets is motivated by the minimization of free energy at the interface. Controlling the interface energy between the three liquid phases is crucial for achieving flexible droplet shape changes. It is foreseen that a wider range of imaginable applications of liquid combinations based on two distinct chemical properties can be developed.

Due to its significant advantages in manipulating the hydrodynamics of micro-droplets, droplet-based microfluidics technology has become a promising method for the preparation of Janus droplets at the (sub)-micron level. The microfluidic device can realize single-step production of high-yield monodisperse Janus droplets, and has great flexibility to control the droplet size and anisotropic shape [11]. In the microfluidic device, each droplet provides a microreactor in which species migration or reaction can occur [12,13,14,15]. Various microfluidic devices, such as cross-flow microchannels, focused fluidics, embedded channels, and co-flow devices, can produce droplets in the microchannel by shearing the dispersed phase in a continuous liquid stream [16,17,18,19,20]. Two mutually adhering immiscible dispersed phase streams enter the microfluidic focusing device, and are sheared into Janus droplets by another stream at the orifice [21,22]. Nisisako et al. used a direct method to provide two-sided star particles for various systems, and explained the advantage of producing highly dispersed two-sided star hemispherical droplets [22,23]. The idea of preparing Janus droplets in a flow-focusing device has been extended to various systems by many researchers [21,24,25]. The size and shape of droplets are controlled by changing the flow rate of the continuous or the dispersed phase. Gupta et al. studied the formation mechanism of droplets in a T-junction channel, and proposed a power-law correlation to predict their size [26]. Wu Ping et al. analyzed the formation of microfluidic bubbles in a cross-junction and the transition mechanism from extrusion to dripping [27]. Wang et al. used a 3-dimensional lattice-Boltzmann method to simulate the formation of Janus droplets in Newtonian fluid in the Y-junction channel and its transition from extrusion to dripping [7]. Fu et al. also proposed the flow ratio and the capillary number (*Ca*) scaling law to estimate the bubble size of different systems [28]. Chen et al. studied the influence of liquid viscosity and surface tension on the formation of droplets, and showed that the slug formation period increases with the increase in surface tension [29]. Li et al. used a fluid-volume method to study the formation of single-phase droplets in a cross-junction microchannel, which was used to guide the design of microchips for droplet formation [30]. Raj et al. used the volume of fluid method (VOF) to study the formation of droplets in T-junction and Y-junction microchannel, and analyzed the influence of flow ratio, liquid viscosity, surface tension, channel size and wall adhesion characteristics on the length of Newtonian fluid slugs [31]. Fu et al. studied the formation of oil droplets in flow-focusing microchannels, and proposed a scaling law for predicting the length of oil droplets [32].

There are many existing reports on Janus droplet/particle preparation methods, but more fundamental studies on underlying mechanism and new phenomena associated can be of great consequence and use, especially those involving non-Newtonian fluids [33,34]. It is well known that the nonlinear rheology in non-Newtonian fluids has a profound influence on the flow dynamics [35]. In biological applications, many fluids exhibit non-Newtonian behaviours, which are amplified in combination with the small length scale in microfluidics. Abate et al. studied the formation of monodisperse particles in a flow-focusing device in a non-Newtonian polymer solution [33]. Arratia et al. reported the thinning of polymer filaments and the rupture of Newtonian and viscoelastic liquids in flow-focusing microchannels [36]. The results show that the decomposition mechanism of Newtonian liquid and polymer liquid with the same viscosity is drastically different. This phenomenon is due to the rheological difference between the two liquids. Qiu et al. numerically studied the droplet formation of non-Newtonian liquids in cross-flow microchannels [37]. From their findings, it is obvious that the rheological parameters of non-Newtonian fluids significantly affect the formation mechanism and size of droplets. Aytouna et al. examined the pinch-off dynamics, yield stress, and shear-thinning fluid of droplets in Newton through experiments [38]. Sontti et al. studied the flow pattern of Newtonian fluid and non-Newtonian fluid using T-junction microchannels [39]. Although the dynamics of droplet breaking in Newtonian fluids has been systematically studied [40,41], there is still lack of in-depth understanding of droplet formation and flow in non-Newtonian fluids at the microscale. In this work, we focus on the shear-thinning of non-Newtonian fluids among other features, and investigate for the first time the microfluidic formation of shear-thinning Janus droplets and their subsequent evolution.

The process of Janus droplet generation in a microfluidic device involves a complex mechanism, which stems from the force competition between surface tension, viscous shear, pressure drop and possible disturbances outside the system. These forces depend on fluid properties, flow geometry and flow conditions. When *Ca* is fixed, the droplet length increases with the increase in *Q*, or the velocity of the dispersed phase liquid increases or the aspect ratio increases [7,42,43]. Garstecki et al. proposed that the droplet size decreases with the increase in *Ca*, and is almost independent of the fluid properties [42]. In addition to the influence of *Ca*, the viscosity contrast after conversion is more important than before. Tice et al. and Sang et al. introduced the viscosity effect to the *Ca*, and announced that more viscous fluid produced greater resistance in the main channel and made the force system unbalanced [44,45]. Sontti et al. obtained a similar power-law of droplet volume from extrusion to dripping [39]. Independent experiments by Xu et al. have also verified these power-law that vary with *Ca* or the flow ratio [46]. Numerical simulations have been successfully used to study the performance of droplet formation in multiphase microfluidics [47,48]. Shardt et al. showed the phenomenon of Janus particle transport in shear flow [49]. Daghighi et al. used a 3D multiphysics model to study the transient motion of Janus droplets in a microchannel [50]. However, the formation of Janus droplets in microfluidic devices has not attracted much attention. The volume-of-fluid (VOF) method is very useful in simulating two-phase flow requiring interface tracking. An adaptive dynamic mesh based on the VOF method further enhances accuracy in capturing the evolution of moving interfaces, and has been used to simulate multiphase flow in microchannels.

Here numerical simulations for the Janus droplet formation and ensuing dynamics in a double Y-junction microfluidic device filled with a shear-thinning fluid are performed by a multiphase model of the OpenFOAM, based on a finite-volume method. The bi-phase VOF is adopted to track the interface with the aforementioned adaptive dynamic mesh. The power-Law model is adopted as a simple constitutive model of the shear-thinning fluid. We describe the effects of rheological properties, surface tension, and velocity ratio on droplet characteristics, including formation mechanism, droplet size, and velocity. These understandings can greatly contribute to controlling the preparation of Janus droplets with non-Newtonian liquids.

## 2. Numerical Simulation and Validation

### 2.1. Governing Equations and Computational Scheme

The open-source CFD software OpenFOAM is used with the multiphase flow solver implemented using the VOF method [51]. Conservation equations are solved for a fluid mixture with distributed concentrations rather than a single fluid for each phase. By solving the transport equation of volume fraction, the interface between the phases is realized. The volume fraction, *α_i_* specifies the volume of a phase in each calculation unit. In a two-phase system, a cell that completely fills one phase is expressed as *α_i_* = 1, and that for the other phase as *α_i_* = 0. Obviously, the interface between the two phases is expressed as 0 < *α_i_* < 1. For a two-phase system, only the volume fraction of one phase needs to be determined because the other is obtained by *α*_2_ = 1 − *α*_1_.

The fluid viscosity and density for each unit are defined as a function of volume fraction as:

(1) $$\eta =\alpha_{CP}\eta_{CP}+\left(1-\alpha_{CP}\right)\eta_{DP}$$

(2) $$\rho =\alpha_{CP}\rho_{CP}+\left(1-\alpha_{CP}\right)\rho_{DP}$$

where *η*_CP_ and *ρ*_CP_ denote the viscosity and the density of the continuous phase, while *η*_DP_ and *ρ*_DP_ denote those of the disperse phase, respectively.

The volume fraction of the entire region is determined by solving the transport equation, which is expressed for *α*_1_ of multiphase flow in OpenFOAM as [52]:

(3) $$\frac{\partial \alpha_{1}}{\partial t}+\nabla \cdot \left(U\alpha_{1}\right)+\nabla \cdot \left(U\alpha_{1}\left(1-\alpha_{1}\right)\right)=0$$

where **U** represents the fluid velocity [53]. For laminar incompressible flow, the conservation of mass and momentum are written as:

(4) $$\frac{\partial \rho }{\partial t}+\nabla \cdot \left(\rho U\right)=0$$

(5) $$\frac{\partial \rho U}{\partial t}+\nabla \cdot (\rho UU)=-\nabla p+\nabla \left(\eta \left(\nabla U+\nabla U^{T}\right)\right)+\frac{\rho \sigma k\nabla \alpha }{\frac{1}{2}\left(\rho_{DP}+\rho_{\mathrm{CP}}\right)}+\rho g$$

where *ρ*, *σ*, *k*, *p*, and **g** represent the fluid density, fluid surface tension, interface curvature, pressure, and gravitational acceleration, respectively.

### 2.2. Flow Geometry

**Figure 1:**
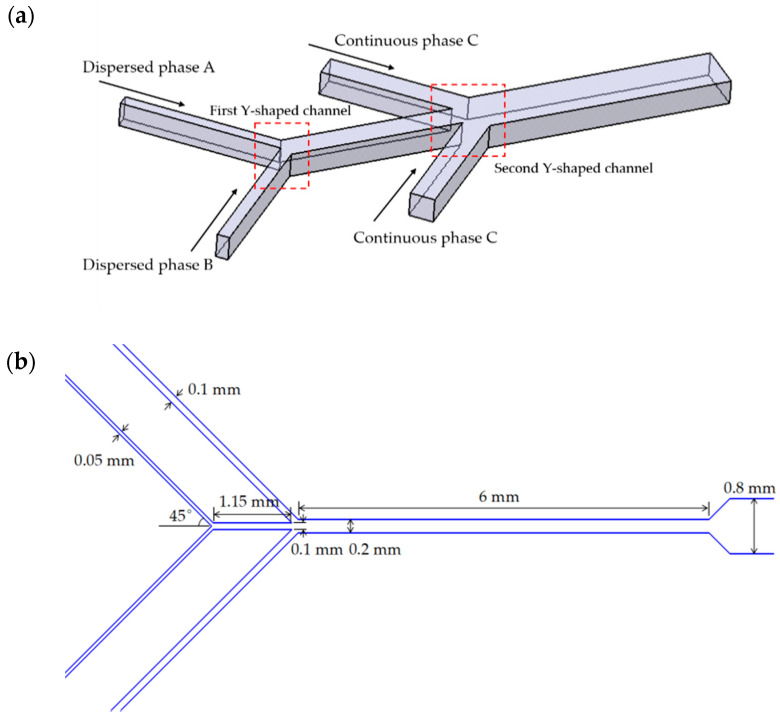
(**a**) Schematic illustration of the double Y-shaped channel used in the manufacture of Janus droplets; (**b**) Specific dimensions of each channel.

A double Y-shaped microfluidic channel is designed to prepare Janus droplets, as shown in Figure 1. The three-dimensional model has a total of four inlets and one outlet. The inlets of the dispersed phase forms a 45° Y-shaped channel. After they merge into one channel, it forms a 45° Y-shaped channel with the other two continuous phase inlets, beyond which Janus droplets are generated and flow through a sufficiently long downstream channel. As shown in Figure 1b, the inlet width of the first Y-shaped channel is 50 μm, and that after the intersection is 100 μm. The inlet width of the second Y-shaped channel is 100 μm, and the width after the intersection is 200 μm, while the depth of this channel is uniformly 100 μm. As shown in Table 1, the two dispersed phase A and B phase are chosen with matching densities, 1,6-hexanediol diacrylate (HDDA) and silicon oil. The two organic phases are incompatible and have an obvious interface. In order to compare differences between Newtonian and shear-thinning fluids, water and 0.25% carboxymethylcellulose (CMC) are selected, respectively. *γ*_ab_ represents the interfacial tension of two dispersed phases, *γ*_ac_ represents the interfacial tension of dispersed phase A and continuous phase C, and *γ*_bc_ represents the interfacial tension of dispersed phase B and continuous phase C. Uniform velocity and pressure are set at each of the four inlets, and the no-slip condition and the contact angle of 165°are imposed on channel walls. The channel upstream to the second Y-shaped channel orifice is filled with A and B phase initially.

### 2.3. Validation

#### 2.3.1. Mesh Independence

**Figure 2:**
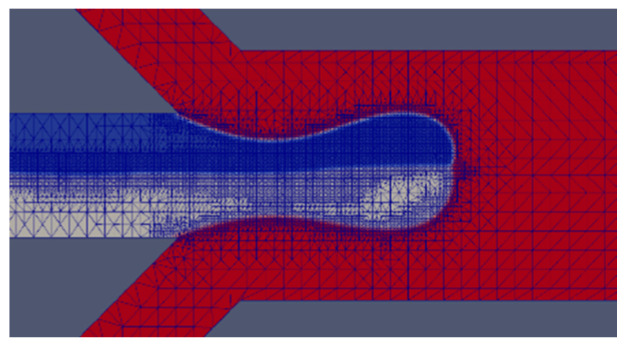
Schematic illustration of adaptive dynamic mesh of Janus droplet at the orifice.

The interface width of a real physical system is much smaller and sharper than the typical diffusive interface thickness adopted in most VOF simulations. Considering the dependence of the VOF method on the mesh size, solution convergence must be ensured with extreme care. The computational reconstruction of the droplet interface is directly related to the reliability of the simulation. The interface capturing method of VOF has a great relationship with the construction of mesh. A suitable mesh can form a clear and thin interface, making the simulation results more reliable. Generally speaking, the denser the mesh, the clearer the interface will be, but excessively dense mesh can also cause divergence. Here an adaptive dynamic mesh is used to refine the two-phase interface of the droplet by changing the maximum number of subdivision layers (maxRefinementInterface) that a cell in OpenFOAM can experience, as shown in Figure 2. Max Refinement (MR) is the maximum number of subdivision levels that a cell can go through. When the refinement level specifies a power of 2, for example, the maximum refinement is 2^−3^ = 0.125 times the original cell size. The subdivision is relative to the size of the current cell, and the typical value is in the range of 2–4. The flow rates of the dispersed and continuous phases are 18 μL/h and 1800 μL/h, respectively, with *Ca*_CP_ = 0.0103 and *We* = 0.051. When the index is equal to 1, although Janus droplets are generated, the interface is very blurred. For MR = 2, the satellite droplets are not obvious; for MR = 3, a clear interface and satellite droplets are formed. In the case of MR = 4 and MR = 5, problematic divergence is encountered as shown in Table 2. Thus, in most computations performed for the parameter values chosen in this study, MR = 3 is employed.

#### 2.3.2. Model Validation

**Figure 3:**
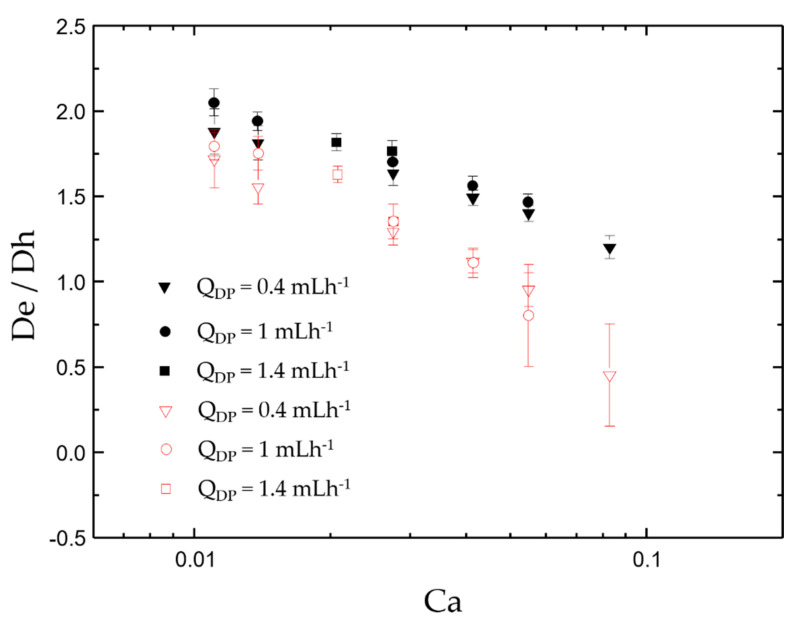
Schematic illustration of error bar of the comparison results of experiment and simulation more comprehensively. The solid icons represent the experimental results, and the open ones are the simulation results.

To validate the numerical model, the numerical results of droplet migration are compared with the experiment result by Nisisako et al. [22]. The standard deviation error bar for three different fixed *Q*_DP_ is studied. At different flow rates, by comparing the characteristic length of the droplets, it is found that the simulation results are consistent with the experimental results. Table 3 shows that the error rate relative to the experiment increases with the increase of the capillary number, *Ca* = *ρ***U**/*γ*, where **U** and *γ* are inlet velocity of continuous phase and surface tension, respectively. In order to show the comparison results of experiment and simulation more comprehensively, error bar is also compared as shown in Figure 3. It can be seen that when *Ca* < 0.04, the droplet diameter is slightly smaller than the reference, but there is no significant difference, and it conforms to the law of change. However, as the number of *Ca* increases, the variance of the Janus droplets generated gradually increases, and it can be seen that the formation of droplets is not stable. Especially, when the *Ca* > 0.1, the change is more obvious. It can be seen that the simulation in this paper is reliable when the *Ca* is less than 0.1.

## 3. Results and Discussion

### 3.1. Phase Diagram

**Figure 4:**
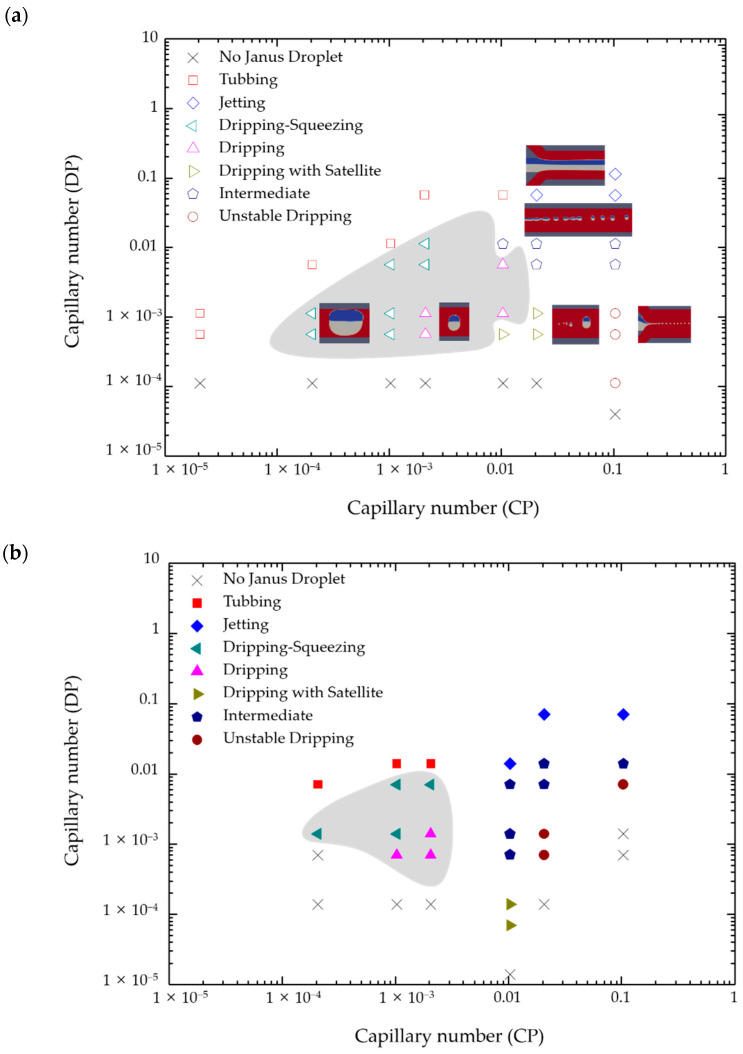
Schematic illustration of the phase diagram. (**a**) The phase diagram in shear-thinning fluid; (**b**) The phase diagram in Newtonian fluid. The gray area represents the dripping state.

The phase diagram of flow states under different *Ca* of the continuous phase and the dispersed phase was obtained, as shown in Figure 4. Diverse working conditions can be obtained by changing the inlet flow ratio. The formation of Janus droplets in the shear-thinning fluid is characterized in five different states: Jetting and tubbing, the fluid neck is not completely contracted and flows downstream in the form of a thread without forming droplets. Jetting has **U**_CP_ much larger than **U**_DP_, and thread width is smaller than the aperture. While tubbing means that **U**_CP_ is much smaller than **U**_DP_, and the thread width is larger than the aperture. Dripping, through continuous phase cutting forms obvious and continuous Janus droplets, which can subdivide into three situations, the Janus droplets are large enough to touch the squeezing of the inner wall of the channel, single dripping droplets and dripping droplets accompanied by satellite droplets. Unstable dripping, the strong force of the continuous phase causes an unstable drip state, which cannot maintain a stable spherical droplet flowing downstream. Intermediate, Janus droplets pinch off an extended thread which maintains the connection with the fluid in the port through the fluid neck at the same time. When the flow rate of the continuous phase further increases, the intermediate state will be a transition to dripping state. Under the situation of a high shear rate, the viscosity of the shear-thinning continuous phase decreases as the number of shear stress increases. Therefore, the viscous stress of continuous phase imposed on the dispersed phase is lower, and the liquid thread will not be pulled downstream further. When *Ca*_DP_ is greater than 0.04, jetting will occur because the large inertial force. When *Ca*_DP_ is less than 0.01, there will be a transition from jetting to dripping with satellite droplets. Inertial force is necessary to induce satellite droplets, because when the viscous force is greater than the inertial force, the formation of droplets will be inhibited; while jetting will appear by the very large inertial force, and the filament will break into Janus droplets further downstream. For the formation of Janus droplets and satellite droplets, the elongated center droplet fluctuates and squeezes at numerous locations then producing a series of minor satellite droplets at a small viscosity ratio. In contrast, when the viscosity is quite prohibitive, the internal flow that causes the rupture is weakened, resulting in a decrease in the formation of satellite droplets.

Viscosity is one of the important factors affecting the formation of Janus droplets. The study of the formation of Janus droplets in non-Newtonian fluids, compared with the Newtonian multiphase system, has important academic and industrial significance. Under the interaction of viscous stress and the surface tension between the two fluids, the formation of Janus droplets can be controlled. The viscous stress of shear-thinning fluid related to the shear rate and the shear rate varies greatly with the change of position. The shear rate has an effect on the viscosity in all directions in the three-dimensional flow. Due to the influence of the shear rate, the viscosity of the solvent becomes smaller, the dispersed phase is more likely to accumulate at the orifice, and the shear force acting on the interface becomes smaller. Under the same conditions, compared with Newtonian fluid, the shear-thinning solution resulting in Janus droplets are larger and have longer intervals.

The control variable method was used to compare the migration of Janus droplets in shear-thinning fluid and Newtonian fluid, where the parameters of each fluid were the same, except for the dynamic viscosity. As shown in Table 4, when *Ca*_DP_ = 0.0007 and 0.0014, the Janus droplet will generate successfully in shear-thinning fluid. However, a series of small Janus droplets were generated in a Newtonian fluid in an intermediate state. When *Ca*_DP_ is greater than 0.07, the frequency of Janus droplets generated in the shear-thinning fluid will be faster due to the effect of smaller viscous force, but the flow state will change from intermediate to jetting in the Newtonian fluid. With the increase of the shear force of the shear-thinning fluid, the viscous force becomes smaller, so that more dispersed phase liquid will inject in the main channel and eventually form larger Janus droplets.

Using an effective model, the formation mechanism and behavior of droplets in non-Newtonian fluids are systematically studied. The following chapters will elaborate on the influence of various rheological parameters, namely the power-law index (*n*), consistency index (*K*), surface tension, and the flow rate of continuous phase (*Q*_CP_) and the flow rate of dispersed phase (*Q*_DP_).

### 3.2. Effect of Power-Law Index and Consistency Index

In this section, the power-law model, *η* = *Kx^n^*^−1^, was chosen to describe shear-thinning fluid. The influence of the consistency index and the power-law index on the formation mechanism, size, and velocity of the droplet is discussed systematically [39].

**Figure 5:**
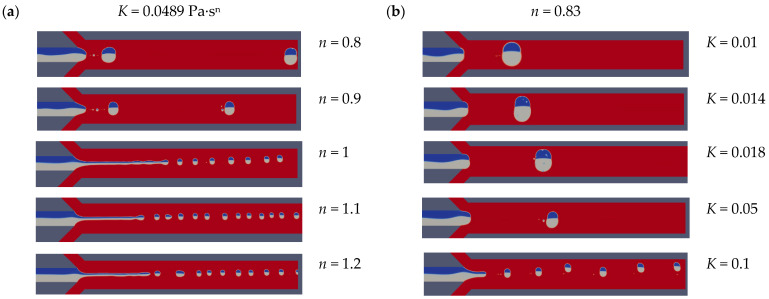
Schematic illustration of the effect of consistency index and liquidity index on droplet length at *Ca*_CP_ = 0.0103, *Ca*_DP_ = 0.0014. (**a**) When *K* = 0.0489 Pa·s^n^, the flow states under different power-law index; (**b**) When *n* = 0.83, the flow states under different consistency index.

In general, the forces acting on the dispersed phases at the cross junction are viscous force, pressure drop, and surface tension. The viscous force is caused by the viscous stress acting on the liquid-liquid interface, which is proportional to the area of the dispersed phase with the velocity gradient. When the *K* value is 0.0489 Pa·s^n^, *n* > 1 represents shear-thickening fluid that shows the intermediate state that the dispersed phase is pulled downstream of the main channel before the droplet ruptures due to the main surface tension as shown in Figure 5a. For *n* < 1, which is shear-thinning fluid, the dripping state that resulting in larger and more stable Janus droplets will be achieved due to the low viscous force. As *n* increases, the effective viscosity of the continuous phase increases, resulting in higher viscous resistance, which helps the rapid separation of droplets. In the case of shear-thinning fluid, surface tension plays a dominant role and delay the separation of dispersed phases at the cross junction. For high-*n* shear-thinning fluid, the droplet length hardly changes. With the increase of *n*, in Newtonian fluids and shear-thickened liquids, due to the increase in viscosity and shear force, droplets are formed quickly before they rise to the top wall of the channel. It can be seen that as the flow state changes from dripping to jetting, the shape of the droplet changes from plug shape to spherical shape.

The generation of Janus droplets in power-law fluids can also be realized by changing the consistency index value. When *n* = 0.83, the various droplets generation state can be obtained in the different continuous phase by changing *K* from 0.01 Pa·s^n^ to 0.1 Pa·s^n^ as shown in Figure 5b. The flow state changes from dripping to intermediate due to the increase in effective viscosity. In addition, the size of Janus droplet decreases with the increase of the consistency index because the interaction between the viscous force and interface force.

In order to realize the influence of the power-law index on the body viscosity and overall viscosity, the effective viscosity of various liquids was estimated from the simulation and compared with the result of Equation (6) [54]:

(6) $$\eta_{\mathrm{eff}}=K{\left(\frac{3n+1}{4n}\right)}^{n}{\left(\frac{8U_{L}}{W_{c}}\right)}^{n-1}$$

**Figure 6:**
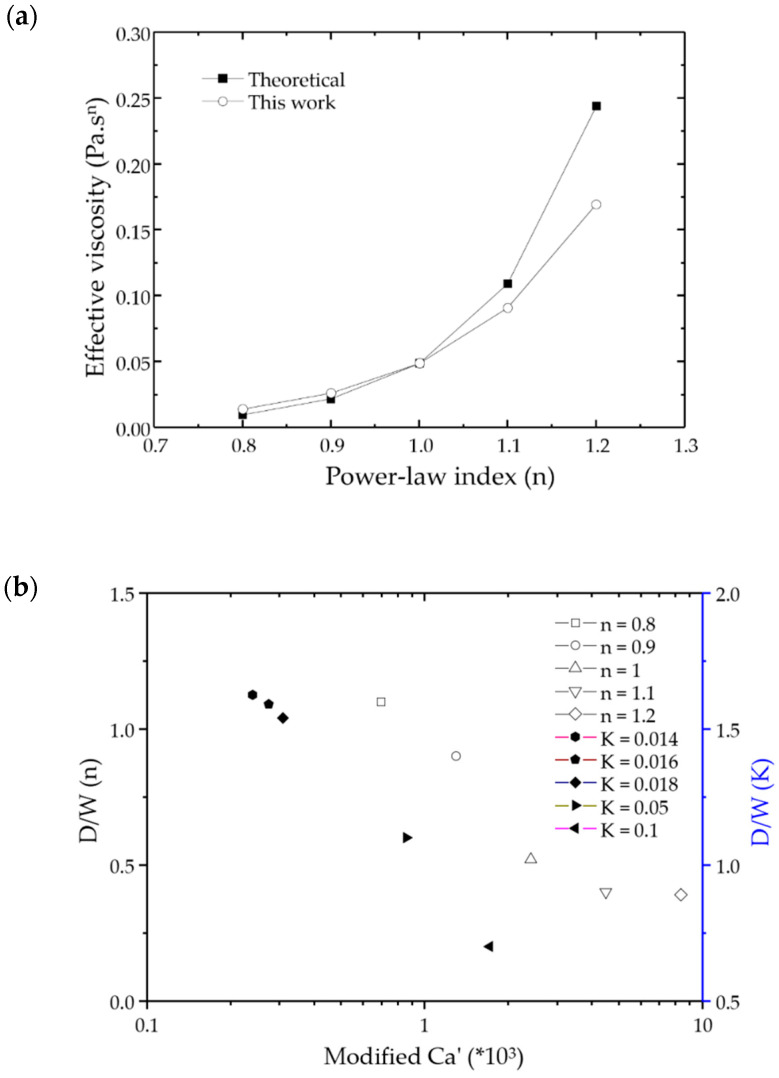
(**a**) Comparison of the calculated result with our simulation result. The solid icons represent the experimental results, and the open ones are the simulation results; (**b**) The scaling relation for the non-dimensional droplet size with the modified *Ca*’ for various power-law liquids at different conditions. The solid icons represent the *K* results, and the open ones are the n results.

As shown in Figure 6a that the effective viscosity increases with the increase of n, and the CFD calculation results agree well with the theoretical values. In the middle of the microchannel, the effective viscosity of the shear-thinning fluid increases, which is the hallmark of the typical non-Newtonian fluid flow characteristics.

The dimensionless droplet size, *D*/*W* where *D* is diameter of Janus droplet and *W* is the width of channel, is scaled using the *Ca*’ as a power-law relationship, as shown in Figure 6b, where *Ca*’ = *K***U**_L_*^n^W*_c_^1−*n*^/*σ*. When *K* = 0.0489 Pa·s^n^, *n* change from 0.8 to 1.2, the size of Janus droplet decrease with the increase of modified *Ca*’. While *n* = 0.83, *K* change from 0.014 to 0.1, it is obvious that the size of the Janus droplet decreases with the increase of modified *Ca*’.

### 3.3. Effect of Surface Tension

**Figure 7:**
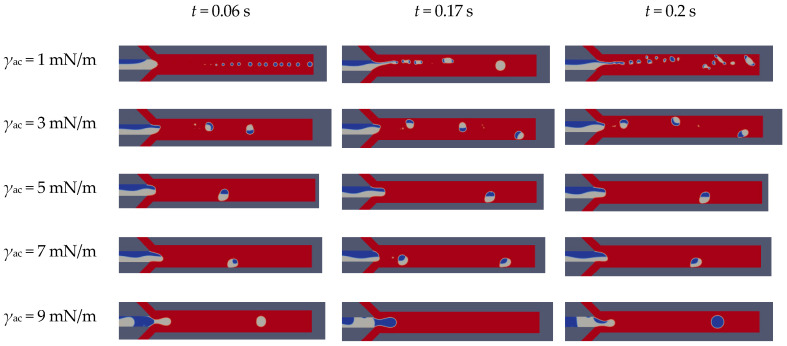
Effect of surface tension on droplet generation at *Ca*_CP_ = 0.0103, *Ca*_DP_ = 0.0014, *γ*_bc_ = 5 mN/m, and *γ*_ab_ = 2.2 mN/m.

In order to understand the effect of surface tension on the formation of Janus droplets, a series of simulations were carried out with additional properties unchanged, where *γ*_ac_ + *γ*_bc_ = 10 mN/m (*S* = *γ*_bc_ − (*γ*_ac_ + *γ*_ab_) < 0). It can be seen from Figure 7 that when *γ*_ac_ (blue) is changed, as the surface tension of the shear-thinning fluid increases, the formation of Janus droplets changes from unstable state to dripping, and finally due to the difference between being increasing and the Janus droplet cannot be formed. As the surface tension of the one side dispersed phase increases to 9 mN/m, the dispersed phase (blue) blocks the front Y-type channel, and its velocity decreases. It can be observed that the size of the Janus droplet increases with the increase of the surface tension. When the surface tension between 3 mN/m and 7 mN/m, the flow state is dripping, and Janus droplets are the most stable when surface tension is 5 mN/m. When the surface tension is 1 mN/m, the phenomena that are more complex can be observed. As the surface tension increases, the satellite droplets will decrease or even disappear. Because the Weber number that determines the inertial force of the droplet is small enough, it can be ignored in this work.

Owing to the change of the curvature of the dispersed phase caused by a continuous phase, the pressure on the droplet increases with the increase of the surface tension. However, the pressure drop over the entire channel length is constant. With the increase of pressure drop on the dispersed phase, the dispersed phase will be cut off faster to form Janus droplets.

### 3.4. Effect of Flow Rate of Continuous Phase and Dispersed Phase

**Figure 8:**
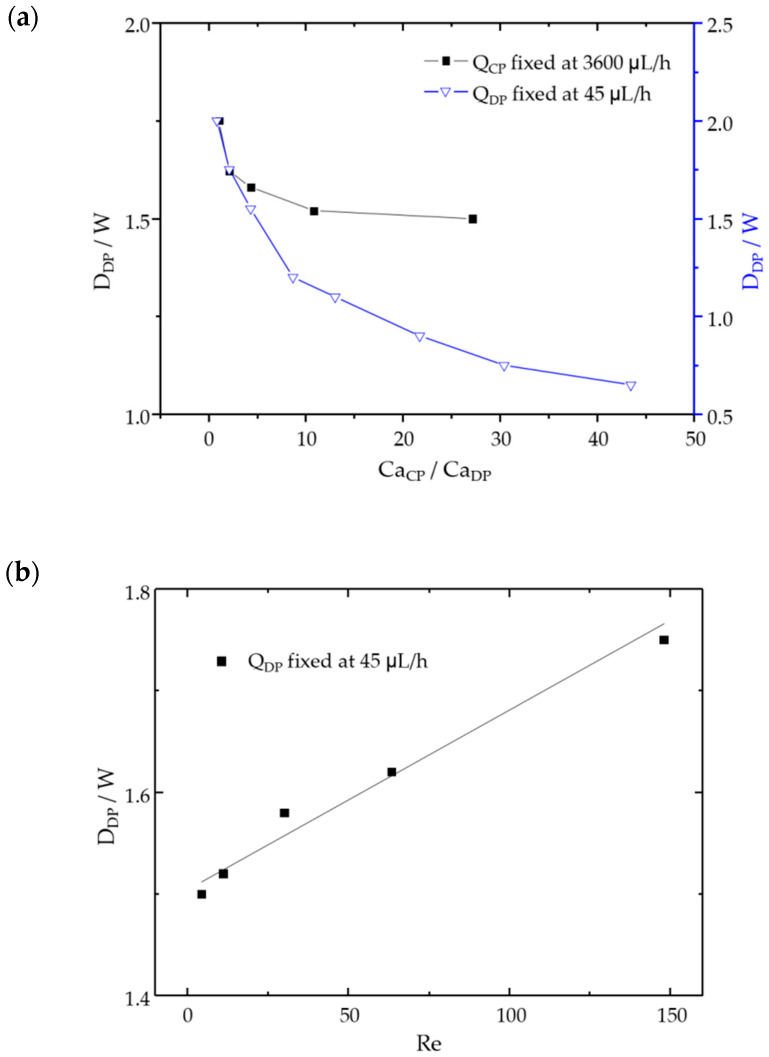
(**a**) Diagram of the characteristic length and *Ca* ratio under different flow rates of continuous phase and dispersed phase; (**b**) Diagram of characteristic length and Re of dispersed phase.

Under the fixed conditions of *K* = 0.048 Pa·s^n^, *n* = 0.83, the influence of *Q*_CP_ on the droplet generation mechanism and size was studied. As the flow rate of dispersed phase *Q*_DP_ = 45 µL/h, the flow rate of continuous phase *Q*_CP_ changed from 720 µL/h to 36,000 µL/h, as shown in Figure 8a. It was observed that the viscosity decreased as the *Q*_CP_ increased, and the flow state changes from dripping to intermediate, and finally reaches unstable dripping. When *Q*_CP_ is small, the resistance of the continuous phase is weak, such that the dispersed phase easily pushes into the main channel and completely blocks at the cross-section. With the shear stress increases, the fluid neck of the dispersed phase gradually decreases, and finally separates when the neck reaches critical thickness. Moreover, as the flow rate of the continuous phase increases, the increase in viscosity and inertial force will cause the droplets to separate quickly in the neck, showing an unstable flow state.

At the condition of *Q*_CP_ = 3600 µL/h, the *Q*_DP_ varies between 7.2–180 µL/h to understand its influence on the droplet generation characteristics. The result can be explained by the Reynolds number of the dispersed phase. It can be observed in Figure 8a that in the case of shear-thinning fluid, with the increase of *Q*_DP_, there is a squeezing state (*D*_DP_/*W* > 1.5). Compared with the Newtonian fluids, due to the increase of the dispersed phases entering the main channel continuously, the flow state changes from a dripping to squeezing with the increase of *Q*_DP_. In the shear-thinning fluid, as the Re of the dispersed phase increases, the droplet size increases significantly, as shown in Figure 8b because the inertial force is sufficient to resist the opposing continuous phase shear stress. However, the shear stress and viscous stress of the continuous phase suppress this inertial effect in Newtonian fluids, making the droplet length change very small.

### 3.5. Mechanism for Janus Droplet Generation

**Figure 9:**
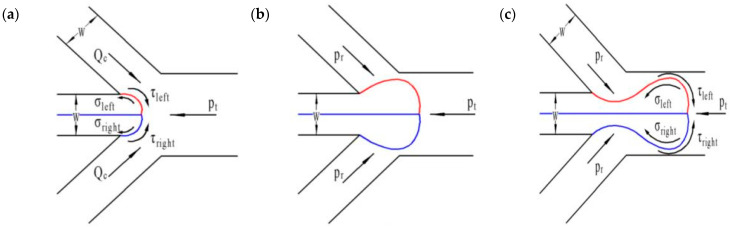
Sketch of emerging droplet prior to detachment and the related force competition. (**a**) Droplet just extruded from the orifice; (**b**) The extruded dispersed phase continuously accumulates at the orifice; (**c**) The dispersed phase was concave toward the centerline under the force of the continuous phase.

For shear-thinning fluid, after the dispersed phase invades the main channel, it grows slowly under the balance of surface tension, shear force, and pressure drop, and is finally pinched to Janus droplets. Before detachment, the schematic diagram of the force on the emerging droplet is shown in Figure 9, which is surface tension, shear stress on the interface, and hydrostatic pressure difference on both sides of the droplet [48]. With the continuous injection of the dispersed phase at the orifice, the pressure gradient on both sides of the droplet in the continuous phase increases under the resistance of surface tension. The pressure caused by the pressure drop is:

(7) $$F_{p}=\left(p_{r}-p_{t}\right)\cdot A_{c}=Q_{c}\frac{k\mu_{c}L_{g}}{h{\left(w-L_{g}\right)}^{3}}L_{g}h=\frac{k\mu_{c}Q_{c}L_{g}{}^{2}}{{\left(w-L_{g}\right)}^{3}}$$

where *Q* = ∆*p*/*R*_hydro_. In shear-thinning fluid, additional Laplace pressure is generated because the arc interface discharges the continuous fluid around itself. It is always affected by the continuous phase on the interface and express as the total pressure along the channel axis. Under the dripping state, the flow velocity between the interface and the wall is large, and the viscous force acting on the interface becomes diluted, the dispersed phase separates quickly by the dual action of surface tension and pressure drop. Viscous shear force can be represented by [55]:

(8) $$F_{\tau }=2\tau \cdot A=2\cos45\mu_{c}\frac{Q_{c}}{h\left(w-L_{g}\right)\left(w-L_{g}\right)}L_{g}h=\frac{\sqrt{2}\mu_{c}Q_{c}L_{g}}{{\left(w-L_{g}\right)}^{2}}$$

where *τ* = *μ*d*u*/d*y* = *μ*d*Q*/*A*d*y*. The surface tension between the two phases is expressed as:

(9) $$\begin{array}{l} F_{\sigma }=\Delta p\cdot A_{c}\\ =\left[\sigma_{1}\left(\frac{1}{h/2}+\frac{1}{L_{g}}\right)-\sigma_{1}\left(\frac{1}{h/2}+\frac{1}{L_{g}/2}\right)\right]\frac{L_{g}}{2}h+\left[\sigma_{2}\left(\frac{1}{h/2}+\frac{1}{L_{g}}\right)-\sigma_{2}\left(\frac{1}{h/2}+\frac{1}{L_{g}/2}\right)\right]\frac{L_{g}}{2}h\\ =-\frac{1}{2}h\left(\sigma_{1}+\sigma_{2}\right) \end{array}$$

In summary, the total force is expressed as:

(10) $$F_{\mathrm{total}}=\frac{\sqrt{2}\mu_{c}Q_{c}L_{g}}{{\left(w-L_{g}\right)}^{2}}-\sigma h+\frac{k\mu_{c}Q_{c}L_{g}{}^{2}}{{\left(w-L_{g}\right)}^{3}}$$

Therefore, under the pressure accumulation and shear of the continuous phase fluid, the formation of droplets is a dynamic process, and the continuous generation is periodic, as are the changes in local pressure and velocity.

### 3.6. Mechanism for Janus Droplet Migration

**Figure 10:**
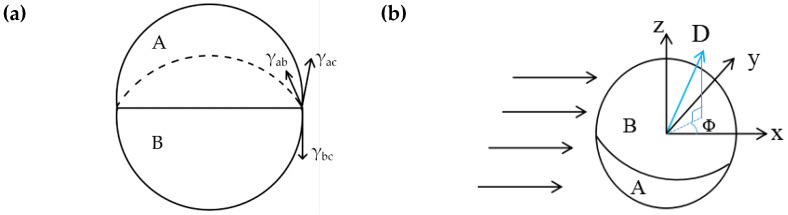
(**a**) 2D illustration of the equilibrium of surface tension in continuous phase; (**b**) 3D illustration of the direction of a Janus droplet.

Janus droplets are formed only within a limited range of *Q*_DP_ = *Q*_A_ + *Q*_B_ at a particular value of *Q*_CP_ under low *Ca*. The three surface tensions can be balanced at equilibrium, as shown in Figure 10a, only if *S* = *γ*_ac_ − (*γ*_ab_ + *γ*_bc_) < 0, where *S* is the spreading parameter. If *S* > 0, the A phase will spread entirely across the B phase to form a core–shell geometry. According to Young’s equation, the surface tension at the interface must satisfy the following relationship to form Janus droplets [56]:

(11) $$\gamma_{\mathrm{ab}}\cos_{\alpha }+\gamma_{\mathrm{bc}}\cos_{\beta }\geq \gamma_{\mathrm{ac}}$$

In a simple shear flow, the direction of Janus droplets can only be characterized by the polar angle *φ*. The analysis here completely ignores the rotational dynamics of the Janus droplet before it reaches its stable direction, because in the case of spherical particles in the expanding flow, regardless of the initial position, they can be aligned with one of the principal axes [55]. The problem can be simply analyzed by looking for the torque-free direction D, as shown in Figure 10b. The process of determining the torque and force on the Janus droplet follows the classic direct method, that is integrating the fluid stress on the outer surface of the droplet. The integration of the surface gravitational force on the surface of the outer droplet (*r* = 1) produces the hydrodynamic force on the Janus droplet:

(12) $$F=\int_{S_{d}}n\cdot \sigma dS_{d}$$

where *S*_d_ is the surface area of the outer droplet. The torque is given by the first moment of surface traction:

(13) $$T=\int_{S_{d}}x\times \left(n\cdot \sigma \right)dS_{d}$$

## 4. Conclusions

The VOF method is used to study the formation and migration of Janus droplets in a double Y-type microchannel using Newtonian fluid/shear-thinning fluid two-phase microsystem. A multiphase fluid flow solver based on the open-source software OpenFOAM, MultiphaseInterDyMFoam, is used to solve the problem. The traditional interface-tracking method VOF depends on the mesh of simulation domain, while more accurate results can be obtained by selecting a dynamic adaptive mesh. A new understanding of the formation process of Janus droplets in non-Newtonian liquids is obtained. Under the shear-thinning fluid, the dynamic characteristics are used to explain the formation and flow state of Janus droplets under different flow conditions. The viscosity on the droplet interface is significantly reduced by the shear-thinning fluids in continuous phase. Compared with the continuous phase of Newtonian fluid under the same environment, the viscosity effect is weaker, resulting in faster separation and a larger droplet size.

In the observed extrusion, jetting, tubing, dripping, unstable dripping and parallel flow under various flow conditions, the power-law index, consistency index, flow ratio, surface tension, and rheology have significant effects on the formation and size of Janus droplets. As the power-law index and consistency index increase, the size of droplet decreases, as the effective viscosity increases. Emulsions produced with non-Newtonian fluids are commonly used for drug delivery and other biochemical applications. Accurate dosage must be ensured for a reliable operation, which requires fine control of Janus droplet size. We have also determined the correlation between the droplet size and the number of flow rate, which may provide a higher degree of control over the Janus droplet size produced by shear-thinning fluids. As the flow rate of the continuous phase increases, the Janus droplet also decreases. On the contrary, the size of the Janus droplet increases with the flow rate of the dispersed phase increases. As with Newtonian media, droplet size increases with the surface tension increases in all cases in non-Newtonian liquids. However, the size of Janus droplets changes from a small bead connected by a liquid thread to a Janus droplet in the case of shear-thinning fluid.

The development of microfluidic methods for generating and manipulating mono-dispersed droplets has brought more potentially interesting applications. Although computational research cannot completely eliminate the necessity of being exhaustive and expensive, a fully verified CFD model can certainly supplement all aspects of physical phenomena that can be obtained by experiments. The present results presented are called upon to provide a better understanding and experimental guidance for the control parameters of forming ideal Janus droplets of different shapes and sizes in non-Newtonian fluid.

## Figures and Tables

**Table 1:** Physical properties of various liquid-liquid systems used.

Phase	Density (kg/m^3^) *ρ*	Dynamic Viscosity (Pa·s) *ν*	Surface Tension (mN/m) *γ*
Aqueous (CP)	1000	0.00101	*γ*_ab_ = 2.2
0.25% CMC (CP)	999.5	0.0489	*γ*_ab_ = 2.8
Silicon oil (DP)	960	0.00934	*γ*_bc_ = 5.3
HDDA (DP)	1000	0.00635	*γ*_ac_ = 4.5

**Table 2:** Results with different max refinement.

Name	Max Refinement	Results
Mesh 1	1	Obscure boundary
Mesh 2	2	The satellite droplet is not obvious
Mesh 3	3	Janus droplet and satellite droplet are both obvious
Mesh 4	4	Interface divergence
Mesh 5	5	Interface divergence

**Table 3:** Comparisons of droplet diameter obtained by the current simulation and previous experiment result.

Parameter	Droplet Diameter (m) *Q*_DP_ = 1.4 mL h^−1^, *Ca* = 0.02	Droplet Diameter (m) *Q*_DP_ = 1 mL h^−1^, *Ca* = 0.04	Droplet Diameter (m) *Q*_DP_ = 0.4 mL h^−1^, *Ca* = 0.1
Empirical value by Nisisako et al.	1.82 × 10^−^^4^	1.5 × 10^−^^4^	1 × 10^−^^4^
Present simulation	1.7 × 10^−^^4^	1.3 × 10^-4^	8 × 10^−^^5^
Rate of deviation	1.07	1.15	1.25

**Table 4:** The difference between Newtonian fluid and shear-thinning fluid under the same conditions.

*Ca*_DP_	0.0007	0.0014	0.007	0.014
Shear-thinning fluid	Janus droplet with satellite	Janus droplet	Janus droplet	Intermediate
	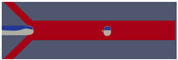	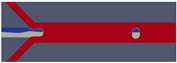	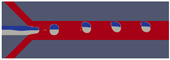	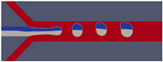
Newtonian fluid	Intermediate	Intermediate	Intermediate	Jetting